# MTA‐TST Axis‐Mediated Apoptosis Activation: A Multi‐Omics Insight Into High‐Protein Diet's Anti‐Adiposity Effect

**DOI:** 10.1002/fsn3.70511

**Published:** 2025-07-09

**Authors:** Xinli Yang, Yueyue Wang, Zhe Shi, Aiting Wang, Jianglan Long, Dan Yan

**Affiliations:** ^1^ Capital Medical University Affiliated Beijing Friendship Hospital State Key Laboratory of Digestive Health National Clinical Research Center for Digestive Disease Beijing China; ^2^ Beijing Institute of Clinical Pharmacy, Beijing Friendship Hospital Capital Medical University Beijing China; ^3^ College of Pharmacy Chengdu University of Traditional Chinese Medicine Chengdu China

**Keywords:** 5′‐deoxy‐5′‐(methylthio)adenosine, apoptosis, mitochondrial function, thiosulfate sulfotransferase gene, visceral adipose tissue

## Abstract

To explore the mechanism of a high‐protein diet (named high protein and rich fat diet, HPRFD) with weight loss effect regulating visceral fat metabolism through endogenous metabolites. Non‐targeted metabonomics compared the spectrum of different metabolites in different groups of experimental mice, and targeted metabonomics examined the target metabolite in visceral adipose tissue (VAT). VAT transcriptomics identified differentially expressed genes. Multi‐mics joint analysis identified target metabolites, genes, and their relationships. Functional annotation revealed shared signaling pathways. 3T3‐L1 adipocytes were treated with metabolites to observe changes in morphology, mitochondrial function, and expression of key genes in the signal pathway. The gene knockdown experiment evaluated the changes in key metabolites in the above functions of cells. Molecular docking predicted metabolite‐protein binding sites. The results showed that 5′‐deoxy‐5′‐(methylthio)adenosine (MTA) was significantly elevated in the HPRFD group (*p* < 0.05). Fecal MTA negatively correlated with TST gene of VAT expression (*r* = −0.90/−0.89). KEGG analysis showed co‐enrichment in apoptosis pathways. HPRFD upregulated TST (1.31‐fold), Bak (6.52‐fold, *p* < 0.01), and Casp‐3 (2.35‐fold, *p* < 0.05) versus HFD. In vitro, 400 μmol/L MTA increased mitochondrial membrane potential (JC‐1 ratio +0.13, *p* < 0.0001) and upregulated TST, Bak, and Casp‐3. The effect of MTA in restoring mitochondrial membrane potential and promoting the expression of Bak and Casp‐3 genes disappeared after TST knockdown. Molecular docking predicted strong MTA‐TST binding (Δ*G* = −1.2 kcal/mol). HPRFD reduced VAT through MTA‐TST‐Bak/Casp‐3 axis, suggesting that MTA has the potential to be developed as a functional substance for obesity prevention and control.

## Graphical abstract

High protein diet reduced visceral adipose tissue through MTA‐TST‐Bak/Casp‐3 axis, suggesting that MTA has the potential to be developed as a functional substance for obesity prevention and control.

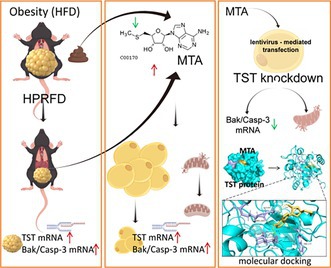

## 1 Introduction

Obesity, as a chronic, recurrent and progressive disease with high incidence worldwide, has become a public health problem that threatens the health of all mankind and brings heavy medical burden (Wang et al. 2021). Obesity is often accompanied by excessive accumulation of VAT, and with the increase of age, the whole‐body fat gradually presents a centripetal distribution, which shows an increase in waist circumference. Central obesity is diagnosed when the waist circumference exceeds a certain range (Yang, Ouyang, et al. 2022). Obesity and central obesity are clearly related to cardiovascular diseases, type 2 diabetes, various types of cancer, premature death, depression, cognitive decline, osteoporosis and weakness (Yang, Bao, et al. 2022), and these diseases are also huge health damage combined with population aging. As far as intervention strategies are concerned, healthy lifestyle including diet, surgery, glucagon‐like peptide‐1 receptor agonists and other drugs are all used for obesity prevention and control (Kadouh et al. 2020), and surgery and drugs often have strict indications and certain adverse reactions (Drucker 2018). The omnivorous nature of human beings determines that compound diet inevitable in obesity promotion and weight control strategies, and plays a vital role, which should be paid more attention than single nutrient (Sievenpiper 2020). At present, popular diet modes such as low‐carbon hydration diet (Meng et al. 2017; Sainsbury et al. 2018), extremely low‐carbohydrate HFD (Kakoschke et al. 2021) and Mediterranean diet (Estruch and Ros 2020) have some limitations (Kakoschke et al. 2021; Yang, Bao, et al. 2022). High‐protein diet, that is, diet with protein energy supply ratio of 20%–35% (Johansson et al. 2014; Schwingshackl and Hoffmann 2013; Yu et al. 2020), has been widely recognized and applied as a recommended diet mode that can effectively reduce weight and maintain weight loss effect (Zhong et al. 2022). In the previous study, we took a high—protein diet as the basis and combined it with the actual intake of dietary fat energy supply ratio of Chinese residents (averaging 33.7% and 36% respectively, data from the Report on Nutrition and Chronic Diseases of Chinese Residents (2020)). Then we designed the high‐protein and rich‐fat diet (HPRFD), which has been proved to have the effects of improving obesity and reducing VAT (Yang, Bao, et al. 2022).

In view of the great harm of excessive accumulation of VAT, we pay more attention to how high‐protein diet reduces VAT. VAT is distributed in the important organs of the abdominal cavity and omentum, and belongs to white adipose tissue. In the past, there were many studies on stimulating brown adipose tissue thermogenesis (Armani et al. 2022; Carpentier et al. 2023; Scheele and Wolfrum 2020), but the study on white adipose tissue was still insufficient. A recent high‐quality study had highlighted that, HFD feeding could fragment the mitochondria of white adipose cells in the groin of male mice. This fragmentation will be aggravated with the increase of HFD intake, and eventually lead to the production of many ineffective and small mitochondria. These mitochondria basically do not burn fat to generate heat, intensifying the process of fat accumulation. In obese individuals, there is also excessive mitochondrion division and fragmentation, which makes those mitochondria that used to work normally lose their functions and accelerates the rate of individual obesity (Xia et al. 2024). This also aroused our curiosity: what substances can promote the metabolism of white fat, and how is the volume and quantity of VAT reduced? With the development of omics technologies (Singh et al. 2023), we have the opportunity to understand the biological “material basis” of various physiological and pathological processes more and more clearly (Jin et al. 2024). More and more evidence showed that diet can play a key role in regulating the body through intestinal flora and metabolites (Benítez‐Páez et al. 2021; Van Meijel et al. 2021). The levels of thousands of metabolites in human plasma metabolomics are strongly influenced by individual heredity, diet and intestinal microbiome composition, and even the formation of healthy metabolomics can be promoted by designing methods to adjust diet or intestinal microbiome (Chen et al. 2022). However, in the past, there were few studies on the mechanism of how high‐protein diet affects VAT metabolism through key metabolites. In addition, many diseases have been proved to be closely related to the abnormal changes of endogenous substances. Discovering new functional substances or new drugs from endogenous active substances has become one of the most important ways (Zhang et al. 2020). We also look forward to looking for functional metabolites to make a preliminary exploration for developing them into functional substances.

## 2 Methods

### 2.1 Experimental Design and Sample Collection

Animal experiment: HFD was used to make an obese mouse model, and HPRFD intervened in the obese mice for 6 weeks. Please refer to our previous research and papers for detailed experimental design and obesity‐related phenotypic results (Yang, Bao, et al. 2022). Visceral adipose tissue: the mice were placed in a supine position, gently lifted the abdominal skin with tweezers, made a small incision in the abdomen, clamped the edge of the skin with tweezers, gently separated the skin with the back of a knife, and pulled the skin to both sides to take out the adipose tissue of the greater omentum. The tissue was fixed and subjected to gradient dehydration, immersion in clearing agents, wax embedding, sectioning, followed by re‐dehydration, staining with oil red O, washing, re‐dehydrating, and finally, sealing the sections under cover slips. The tablets to be sealed were observed under the microscope after drying, and the cell morphology, tissue structure, and pathological changes were recorded. Cell experiment: An obese adipocyte model in vitro was used, and the changes in cell morphology, fat droplets, and mitochondrial function were observed after intervention with different concentrations of MTA. This study used a 3T3‐L1 mouse embryonic fibroblast cell line to induce adipogenesis and establish an obese adipocyte model. After 4 weeks of induction, a large number of lipid droplets were stained with oil red O. After judging the success of induction, the cells were intervened with low, medium, and high concentration gradient MTA according to the intervention concentration and duration provided in previous studies (Moreno et al. 2014). Cells were treated with MTA at low (100 μmol/L), medium (200 μmol/L), and high (400 μmol/L) concentrations. Four groups were set up: the model group, the MTA 100 μmol/L group, the MTA 200 μmol/L group, and the MTA 400 μmol/L group.

After treatment, cells from each group were collected for morphological observation and functional assays. The detection content included JC‐1 staining for mitochondrial membrane potential, ATP detection, ROS staining, and quantitative real‐time polymerase chain reaction (RT‐qPCR) analysis of the mitochondrial gene TST and key apoptosis protein genes Bak and Casp‐3. For RT‐qPCR, one sample per group of cells was sent for analysis, with technical replicates performed three times. Statistical analysis was then conducted to compare differences between the groups.

### 2.2 Laboratory Testing and Statistical Analysis

Following the intervention, cells from each group were collected for morphological observation and functional assessments. Mitochondrial membrane potential was evaluated via JC‐1 staining, while cellular ATP levels and reactive oxygen species (ROS) were quantified. RT‐qPCR was performed to analyze mitochondrial gene TST and apoptosis‐related proteins Bak and Casp‐3. Each experimental group contributed one sample for qPCR analysis, with technical triplicates. Statistical comparisons were conducted to evaluate intergroup differences.

### 2.3 Untargeted Fecal Metabolomic Profiling

Thirty fecal samples from three experimental groups underwent untargeted metabolomic analysis using liquid chromatography‐mass spectrometry, as previously described (Yang, Bao, et al. 2022). Metabolites displaying significant intergroup differences (Student's *t*‐test, *p* < 0.05) were prioritized for pathway enrichment analysis. Over‐representation analysis was employed to identify KEGG species‐specific pathways enriched with these metabolites (*p* < 0.05). Metabolite annotation utilized the KEGG. Data visualization and statistical analyses were performed using R and GraphPad Prism 10. One‐way ANOVA with Bonferroni correction was applied for multi‐group comparisons, with significance set at *p* < 0.05.

### 2.4 Visceral Adipose Tissue Transcriptomics

Libraries were sequenced on the Illumina NovaSeq platform (150 bp paired‐end reads). Raw data were processed via BMK Cloud (www.biocloud.net), including quality filtering, reference genome alignment (Hisat2), and transcript quantification (Fragments per Kilobase Million, FPKM). Differentially expressed genes (DEGs) were identified using DESeq2 (|log2FC| ≥ 1.5, FDR‐adjusted *p <* 0.01). Functional annotation leveraged Nr, Pfam, EuKaryotic Orthologous Groups/Clusters of Orthologous Groups of proteins Swiss‐Prot, KEGG, and Gene Ontology databases. Pathway enrichment was assessed via KEGG Orthology Based Annotation System and cluster Profiler.

### 2.5 Multi‐Omics Integration

UV‐scaled metabolomic and transcriptomic datasets were integrated using two‐way orthogonal partial least squares (O2PLS) to evaluate cross‐modal correlations. High‐loading genes/metabolites were prioritized for network analysis. Fisher's exact test with Bonferroni correction (*p <* 0.05) identified enriched pathways. Enrichment factors were calculated as:

$$\text{Rich Factor}=\left\{\frac{\mathrm{DEG}\;\text{in Pathway}}{\mathrm{DEG}\;\text{in}\;\mathrm{All}\;\text{Pathways}}\backslash \text{over}\left\{\frac{\mathrm{All}\;\text{Gene in Pathway}}{\mathrm{All}\;\text{Gene in}\;\mathrm{All}\;\text{Pathways}}\right\}\right\}$$

### 2.6 Visceral Adipose and Adipocyte RT‐qPCR

RNA extraction, reverse transcription, qPCR (Bustin et al. 2009), and primer design (Chen et al. 2023) followed standard protocols. Primer sequences were listed in Table 1.

**TABLE 1:** RT‐qPCR detection of housekeeping gene and primer sequence information of target gene in visceral adipose tissue and obese adipose cells of mice.

Gene		Primer	Sequence
mGAPDH	Internal reference gene 1 (mouse)	mGAPDH‐F	GGTTGTCTCCTTCA
		mGAPDH‐R	TGGTCCAGGGTTTCTTACTCC
	Internal reference gene 1 (mouse)	m18s‐F	GGCCGTTCTTAGTTGGTGGAGCG
		m18s‐R	CTGAACGCCACTTGTCCCTC
	Internal reference gene (cell)	mGAPDH‐F	GTCAAGGCCGAGAATGGGAA
		mGAPDH‐R	CTCGTGGTTCACACCCATCA
mBak		mBak‐F	CAGCTTGCTCTCATCGGAGAT
		mBak‐R	GGTGAAGAGTTCGTAGGCATTC
mTST		mTST‐F	TGGCGGAATCCATTCGGTC
		mTST‐R	TCATAAGGCGAAGTCGTGTCC
mCasp‐3		mCasp‐3‐F	AGCAAGTCAGTGGACTCTGG
		mCasp‐3‐R	CAGAGCGAGATGACATTCCAGT

According to the experimental data of RT‐qPCR, the average gene content in each cell needs to be obtained by calculating the CT value of the gene. When there was one reference gene, 2^−ΔΔCT^ method (Livak and Schmittgen 2001) was used to calculate. When there were two internal reference genes, the following formula shall be used for calculation:

$$\text{Relative gene expression}=\frac{{\mathrm{RQ}}_{G0I}}{\text{Geomean}\left[{\mathrm{RQ}}_{\text{REFs}}\right]}$$

### 2.7 Demonstration of TST Function by Gene Editing Experiment

The 3T3‐L1 preadipocytes were thawed, cultured to confluence, and subsequently divided into three experimental groups: wild‐type control group (WT): untreated parental 3T3‐L1 cells maintained under standard culture conditions without any genetic manipulation; model group: lentivirus‐mediated stable TST‐knockdown 3T3‐L1 adipocytes generated through RNA interference, followed by adipogenic differentiation to establish the obese adipocyte model; MTA‐treated group (model + MTA): identical to the model group but additionally treated with 400 μmol/L MTA for 24 h after differentiation. At the experimental endpoint, cells from all three groups were subjected to: RT‐qPCR analysis to quantify mRNA expression levels of apoptosis‐related genes (Bax, Bcl‐2, Caspase‐3) and JC‐1 staining to assess mitochondrial membrane potential (ΔΨm) alterations. The methods of adipogenesis induction, RT‐qPCR detection and JC‐1 detection are the same as those in the previous experiments.

### 2.8 Prediction of Binding Sites Between MTA and TST Protein by Molecular Docking

Molecular docking of small molecular metabolite MTA and TST protein was carried out. The crystal structure of the protein was obtained and established by the AlphaFold database (https://alphafold.ebi.ac.uk/) and the docking small molecule database PubChem database (https://pubchem.ncbi.nlm.nih.gov/). Firstly, the protein crystal structure was dehydrated and hydrogenated by AutodockTools5.6, and the acceptor structure was prepared. Use OpenBabel and AutoDock programs to split the small molecule library and perform other preparatory work. The docking is carried out by using the AutoDock program, and finally, the results are imported into PyMOL to visualize the docking results.

## 3 Results

### 3.1 Fecal Metabolomics Identifies MTA as a Key Regulator

**FIGURE 1:**
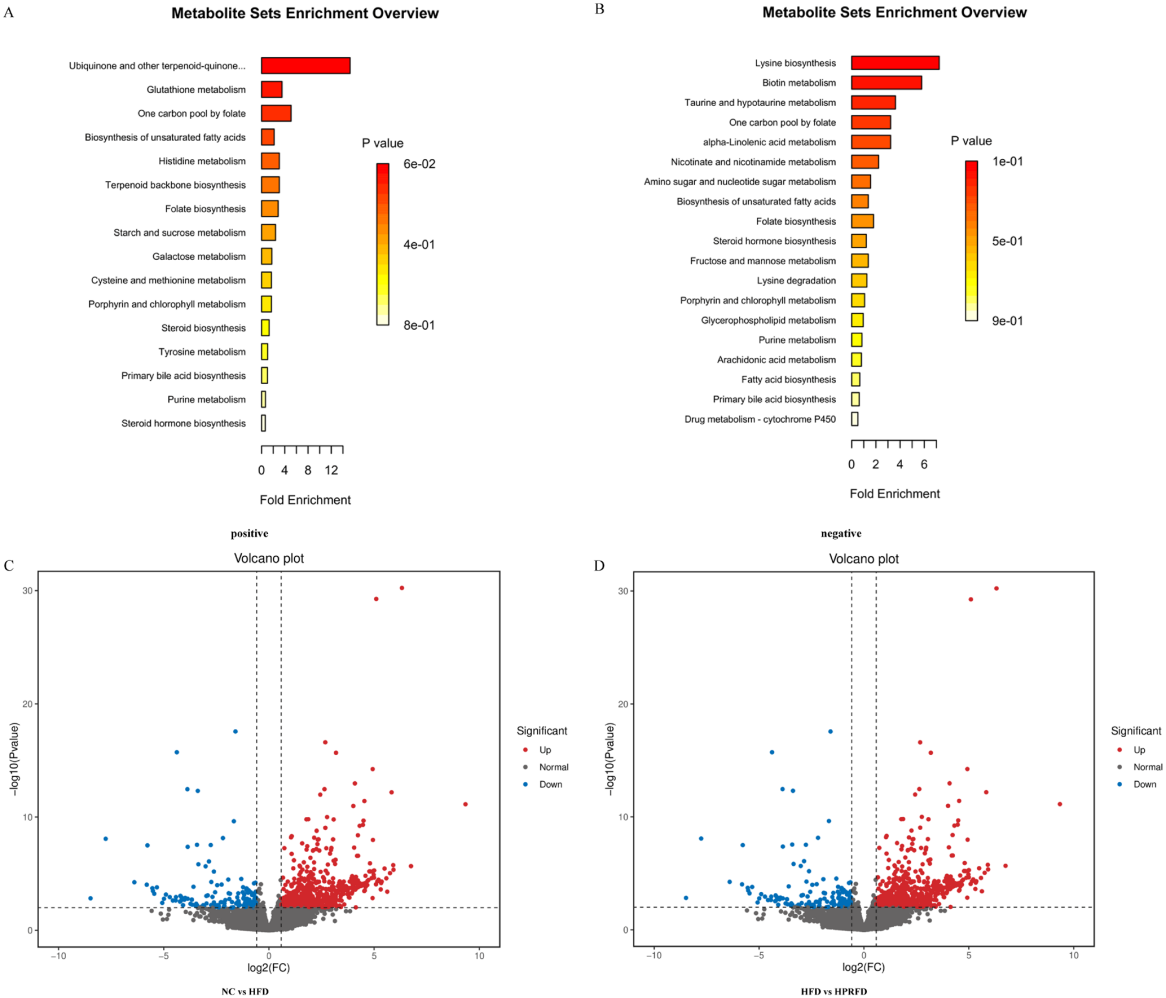
Metabolomics‐transcriptome detection and combined analysis results. (A, B) Analysis diagram of differential metabolite ORA enrichment in mouse feces by non‐targeted metabolomics detection. The abscissa is the enrichment multiple, which is the number of observed metabolites/theoretical metabolites in the metabolic pathway. The size of the *p*‐value is indicated by color, and the darker the color, the smaller the *p*‐value; (C, D) volcano plots of VAT transcriptomes differential gene expression between groups. Points represent genes: Blue (downregulated), red (upregulated), gray (non‐significant). Axes: log_2_(FC) vs. −log_10_(*p*‐value). Dot size/color indicate pathway enrichment significance (the bigger and redder, the more significant the difference is).

Untargeted metabolomic analysis of fecal samples revealed distinct metabolite profiles across experimental groups (qualitative and quantitative results of metabolites: negative 678, positive 1039). Moreover, pathway enrichment highlighted cysteine/methionine metabolism as one of the main differential metabolic pathways (Figure 1A,B).

Among many different metabolites (Appendix S1: Table S1), MTA is the main metabolite related to protein metabolism. It was upregulated in NC vs. HFD and downregulated in HPRFD vs. HFD, establishing MTA as the focal metabolite.

### 3.2 Visceral Adipose Tissue MTA Accumulation

**FIGURE 2:**
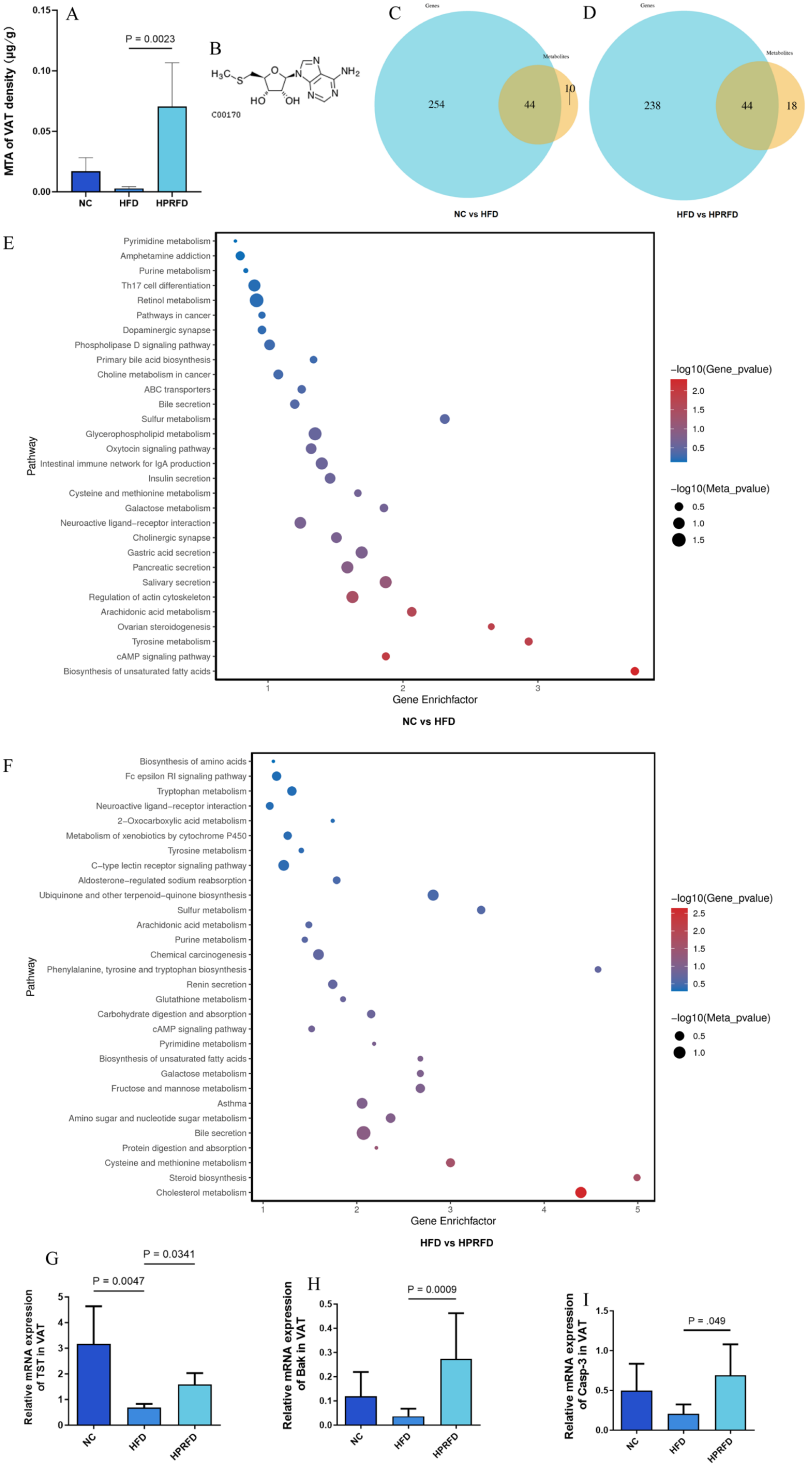
Detection of VAT targeted metabolomics and joint analysis of fecal non‐targeted metabolomics and VAT transcriptome. (A) Targeted metabolism of visceral adipose tissue detected the protein‐related metabolite MTA, which showed that it was down‐regulated in the HFD group, while HPRFD was significantly up‐regulated; (B) MTA structural formula; (C, D) joint analysis of fecal non‐targeted metabonomic and VAT transcriptome Venn diagram. Blue circles represent transcriptome and yellow circles represent metabolomics; the values in the circles represent the number of common/unique metabolic pathways of the two omics. The sum of all the numbers in the circles represents the total number of pathways in which the transcriptome and metabolomics participate, respectively, and the overlapping area of the circles represents the number of KEGG pathways in which both genes/metabolites identified by the two omics participate; (E, F) results of KEGG enrichment analysis. The KEGG‐enriched bubble charts of the first 30 pathways of mouse VAT transcriptome were compared between NC vs. HFD and HFD vs. HPRFD. The abscissa represents the enrichment factor (Diff/Background) of this pathway in different omics, and the ordinate represents the name of the KEGG pathway. The gradual change of red‐blue represents the significant change of enrichment from high to low, expressed by *p*‐value; the shape of the bubbles represents different omics, with the circle representing the transcription group and the triangle representing the metabolism group; the bubble size represents the number of differential metabolites or genes, with a larger number indicating a larger point; (G) the results of mitochondrial TST gene RT‐qPCR detection showed that compared with the HFD group, the TST gene in the HPRFD intervention group was adjusted back, and the difference was significant; (H, I) the RT‐qPCR detection results of Bak/Casp‐3 genes, which showed that compared with HFD, the key genes of apoptosis were significantly increased in the HPRFD intervention group.

Targeted quantification confirmed MTA depletion in HFD visceral adipose (decreased 5.98‐fold vs. NC, *p <* 0.05), with HPRFD restoring levels to 25.1‐fold vs. HFD, *p <* 0.05; Figure 2A.

MTA, the structural formula was showed in Figure 2B, the chemical formula is C_11_H_15_N_5_O_3_S, and the molecular weight is 297.33 Da.

MTA was enriched in visceral adipose tissue after HPRFD intervention, which supports us to continue to explore whether MTA is related to the change of tissue gene expression and whether it can exert positive biological effects on visceral adipose tissue or cells. So, we carried out the transcription group of visceral adipose tissue and cell experiments in vitro.

### 3.3 Transcriptomic Signatures in Visceral Adipose

RNA sequencing identified 700 and 431 DEGs in NC vs. HFD and HFD vs. HPRFD comparisons, respectively. In the NC vs. HFD comparison, the number of DEGs was 820, with 673 up‐regulated genes and 147 down‐regulated genes. In the HFD vs. HPRFD comparison, the number of DEGs was 519, with 131 up‐regulated genes and 388 down‐regulated genes.

KEGG classification revealed that there were many biological processes related to differential genes (Figure 2E,F). These results also reflected the complexity of the influence of compound diet intervention on metabolism.

### 3.4 Cross‐Omics Analysis Links MTA to Mitochondrial Apoptosis

Integrated metabolomic and transcriptomic analyses showed that in visceral adipose tissue, O2PLS integration of 254/238 DEGs and 10/18 metabolites (NC vs. HFD/HFD vs. HPRFD) mapped 44/44 shared pathways (Figure 2C,D).

To elucidate key regulatory mechanisms, we conducted cross‐comparisons of overlapping metabolic pathways, DEGs, and metabolites between groups. Correlation analyses further explored potential gene‐metabolite relationships. The non‐targeted fecal metabolomics and visceral adipose transcriptome datasets provided a robust foundation for identifying MTA related genes through systematic comparisons of NC vs. HFD and HFD vs. HPRFD groups Table 2.

**TABLE 2:** The common differential metabolic pathways, genes, and metabolites between two groups were compared.

Substance metabolism	Pathway	Gene	Metabolote
Carbohydrate metabolism‐related	Galactose metabolism	ENSMUSG00000025877 ENSMUSG00000032401	Dulcitol galactitol, dulcite
Protein metabolism‐related	Cysteine and methionine metabolism	ENSMUSG00000030268 ENSMUSG00000044986	MTA
Fat metabolism related	Arachidonic acid metabolism	ENSMUSG00000003484 ENSMUSG00000029919 ENSMUSG00000029925 ENSMUSG00000030483 ENSMUSG00000051855	
	Biosynthesis of unsaturated fatty acids	ENSMUSG00000021228 ENSMUSG00000042540	Eicosapentaenoic acid

MTA correlated inversely with TST (ENSMUSG00000044986; Spearman *p* = −0.94, *p* = 0.056; Table 3).

**TABLE 3:** Transcription‐metabonomics association analysis of differential genes related to MTA and its related metabolic pathways in KEGG database.

Ensembl ID	Gene ID	Symbol	Description	Type_of_gene	Metabolic pathway
ENSMUSG00000030483	13088	Cypb10	Cytochrome P450, family 2, Subfamily b, polypeptide 10	Protein‐coding	Arachidonic acid metabolism; Retinol metabolism
ENSMUSG00000063558	11761	Aox1	Aldehyde oxidase 1	Protein‐coding	Retinol metabolism
ENSMUSG00000044986	22117	TST	Thiosulfate sulfurtransferase, mitochondrial	Protein‐coding	Cysteine and methionine metabolism; Sulfur metabolism
ENSMUSG00000025037	17161	Maoa	Monoamine oxidase A	Protein‐coding	Arachidonic acid metabolism; Serotonergic synapse
ENSMUSG00000020654	104111	Adcy3	Adenylate cyclase 3	Protein‐coding	Thermogenesis
ENSMUSG00000031659	11513	Adcy7	Adenylate cyclase 7	Protein‐coding	Thermogenesis; Purine metabolism; Bile secretion; cAMP signaling pathway
ENSMUSG00000040907	232975	Atp1a3	ATPase, Na+/K+ transporting, alpha 3 polypeptide	Protein‐coding	Bile secretion
ENSMUSG00000042476	18670	Abcb4	ATP‐binding cassette, sub‐family B member 4	Protein‐coding	ABC transporters
ENSMUSG00000035283	11554	Adrb1	Adrenergic receptor, beta 1	Protein‐coding	Neuroactive ligand‐receptor interaction; cAMP signaling pathway; Pyrimidine metabolism
ENSMUSG00000040016	19218	Ptger3	Prostaglandin E receptor 3 (subtype EP3)	Protein‐coding	Pyrimidine metabolism; cAMP signaling pathway; Neuroactive ligand‐receptor interaction

The correlation coefficients of MTA and TST gene were −0.90 (NC vs. HFD) and −0.89 (HFD vs. HPRFD), respectively.

### 3.5 Signal Pathway Enrichment Analysis of MTA and TST Gene

Screening possible signal pathways by functional annotation of MTA and TST genes in KEGG database. The results showed that they were annotated as the following signal pathways: MTA KEGG_pathway_annotation includes Autophagy–animal (ko04140), Lysosome (ko04142), Apoptosis (ko04210) and NOD‐like receptor signaling pathway (ko04621). TST KEGG_pathway_annotation includes MAPK signaling pathway (hsa04010), Apoptosis (hsa04210), Antigen processing and presentation (hsa04612), Toll‐like receptor signaling pathway (hsa04620), receptor signaling pathway (hsa04621) and JAK–STAT signaling pathway (hsa04630).

It could be seen that KEGG pathway mapping localized MTA and TST to overlapping apoptotic and NOD‐like receptor signaling cascades.

TST is a mitochondrial gene. Mitochondria are organelles that maintain cell biological energy by producing ATP, and are also components of metabolic precursor synthesis, calcium regulation, reactive oxygen species production, immune signal, and apoptosis (Harrington et al. 2023). The mitochondrial pathway dependent on mitochondria is one of the important pathways of apoptosis (Che et al. 2022). We employed RT‐qPCR to validate the expression patterns of mitochondrial TST and apoptosis regulators Bak/Casp‐3, while investigating MTA's modulatory effects. Our results revealed that HPRFD upregulated adipose TST (1.31‐fold, *p* > 0.05), Bak (6.52‐fold, *p* < 0.01) and Casp‐3 (2.35‐fold, *p* < 0.05) versus HFD group (Figure 2H–J). These expression dynamics exhibited similar change with MTA concentrations in visceral adipose tissue, suggesting MTA's potential role in mitochondrial apoptosis regulation.

Correlation analysis of fecal metabolomics and VAT transcriptome revealed mitochondrial TST expression negatively correlated with MTA levels (*r* = −0.90/−0.89). KEGG analysis demonstrated their co‐enrichment in apoptosis pathways.

In the subsequent cell experiments, we tested the mitochondrial function of obesity model cells after MTA intervention to find out whether MTA can affect the mitochondrial function of obese adipocytes.

### 3.6 RT‐qPCR Detection of Visceral Adipose Tissue Verifies the Expression of TST Gene and key Protein Gene of Apoptosis Pathway

RT‐qPCR detection results showed that TST gene and Bak/Casp‐3 gene in visceral adipose tissue of mice were decreased in the HFD group, and they were adjusted back in the HPRFD group, which was consistent with the change trend of MTA content in visceral adipose tissue (Figure 2H–J).

## 4 Obese Adipocyte Model Verified the Intervention Effect of MTA on Adipocytes

### 4.1 Adipocyte Model of Adipogenesis Induced by 3T3‐L1 Cell Line

The effect of inducing adipogenesis was observed under a microscope, and images were collected for evaluation. After 4 weeks of induction, it was observed that the number and size of lipid droplets no longer increased, and the induction was considered successful. After oil red O staining, the lipid droplets appeared red or orange. The induction process and oil red staining results after induction were shown in supplementary material S2.

### 4.2 Different Concentrations of MTA Were Used to Intervene in Obese Adipocytes to Observe the Cell Changes

#### 4.2.1 Morphological Changes of Cells

**FIGURE 3:**
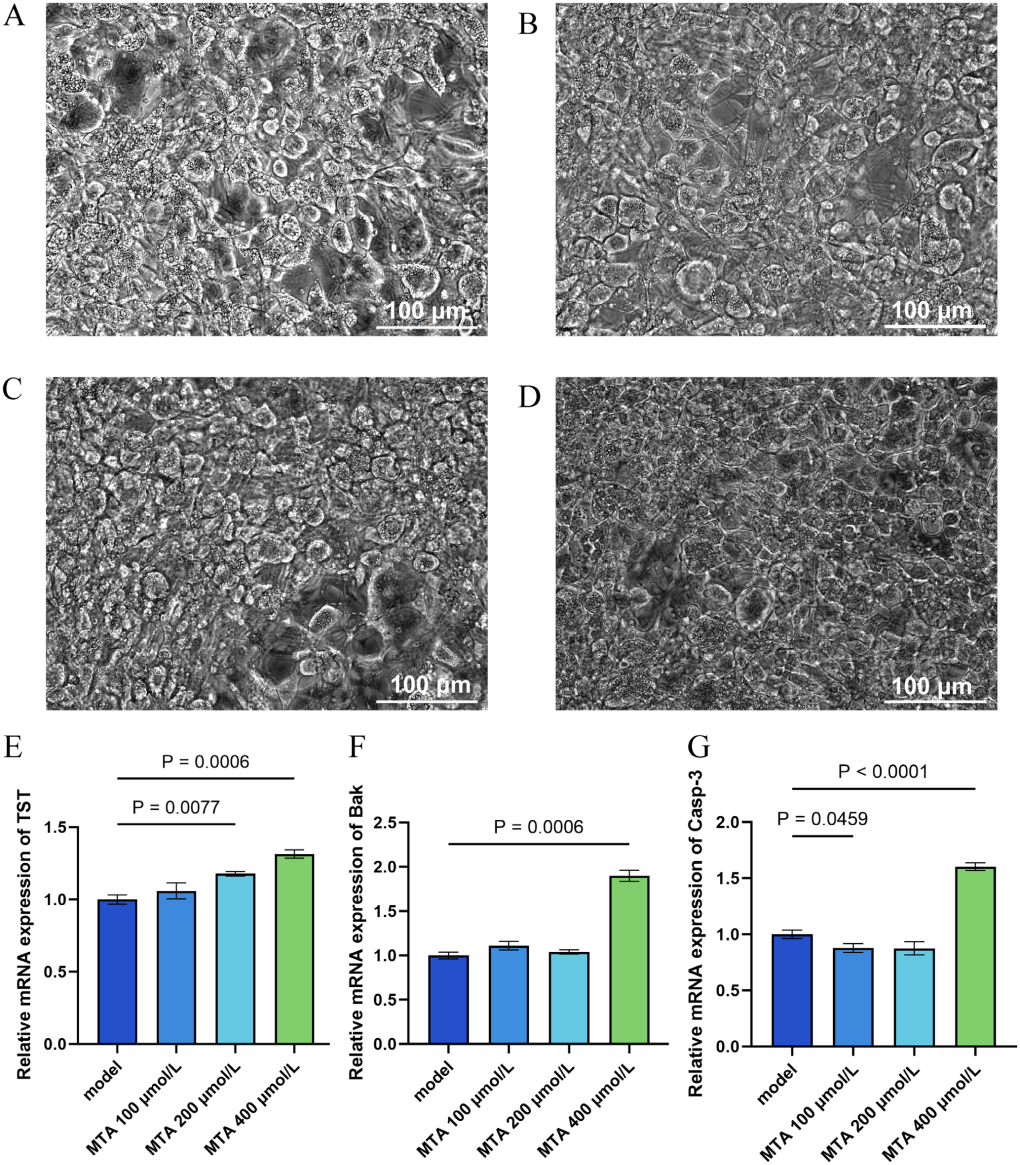
The results of microscope imaging and mRNA expression of obese adipocytes were intervened by MTA with different concentrations. (A–D) 200× microscope field of view of obese adipocytes in the model group and MTA intervention group with different concentrations; the results showed that compared with the model group, the obese adipocytes in the visual field of the MTA 200 μmol/L and 400 μmol/L groups decreased slightly, and the fat droplets decreased slightly; (E–G) detection chart of the mRNA expression level of OAS2 gene among cells in each group. In this experiment, four groups of cells were used to detect the expression levels of three genes, namely Bak, Casp‐3, and TST. From the detection results of the three genes, the expression of all three genes was the highest when the concentration of MTA was 400 μmol/L, compared with the model group.

Observing the changes of cells in different groups under a 200 × microscope, as shown in Figure 3A–D.

The results showed that compared with the model group, the obese adipocytes in the visual field of the MTA 200 μmol/L and 400 μmol/L groups decreased slightly, and the fat droplets decreased somewhat. The MTA 100 μmol/L group did not change significantly.

#### 4.2.2 RT‐qPCR Action Verified the Expression of the TST Gene and Apoptosis Key Protein Gene

The mRNA expression levels of the OAS2 gene in each group of cells were shown in Figure 3E–G. Based on the results of the three genes, 400 μmol/L MTA upregulated TST (1.31‐fold), Bak (1.9‐fold), and Casp‐3 (1.6‐fold) expression versus the model group (*p <* 0.0001). This suggested that the 400 μmol/L concentration of MTA might play a crucial role in regulating the internal energy metabolism of obesity model cells.

#### 4.2.3 Detection of Mitochondrial Membrane Potential by JC‐1 Staining

**FIGURE 4:**
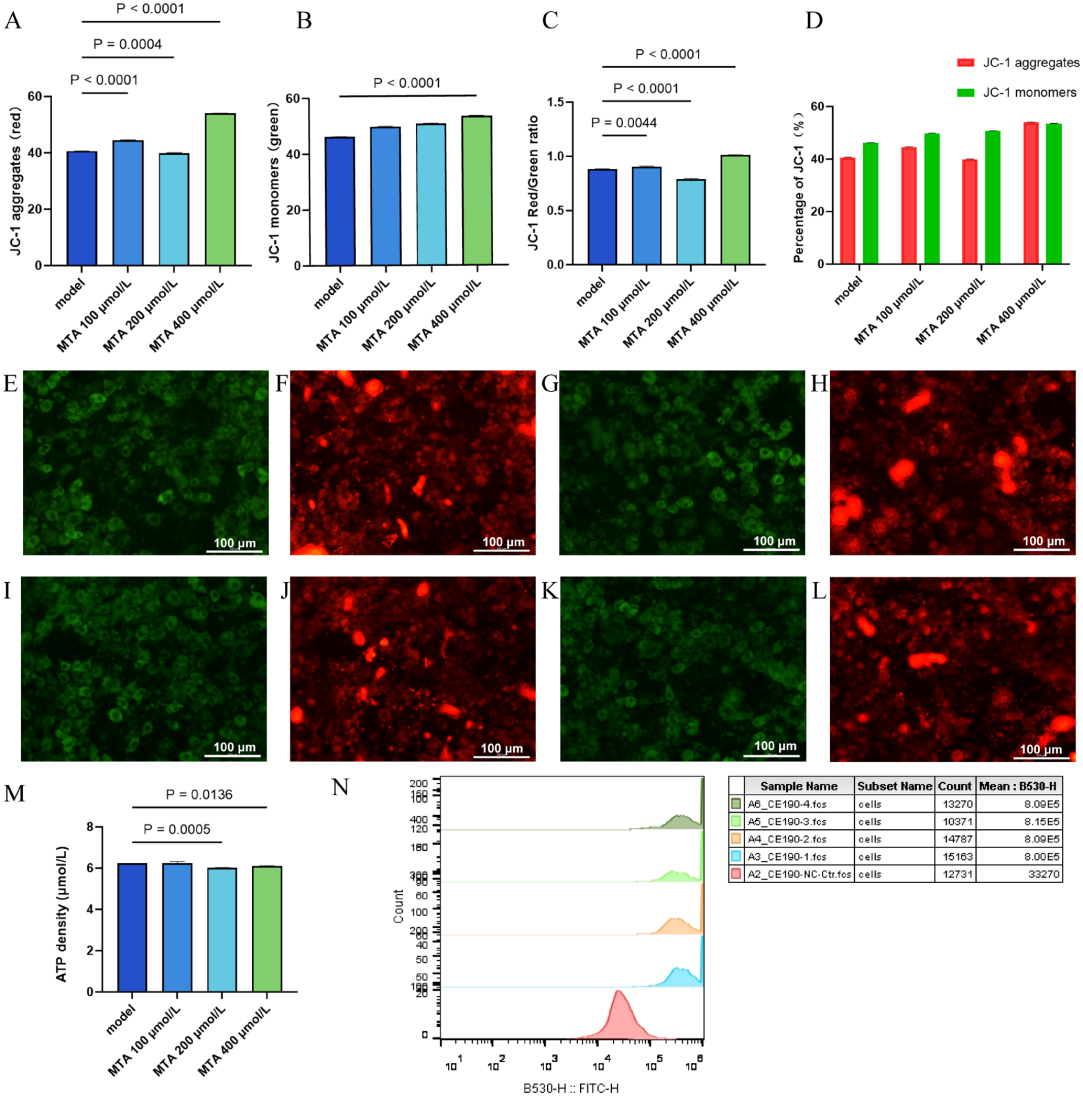
The obesity adipocyte model verifies the intervention effect of MTA on adipocytes. (A–D) The results of JC‐1 staining by flow cytometry after the intervention of adipocytes with different concentrations of MTA; (A) the changes of red fluorescence intensity in each intervention group compared with that in the model group. It increased in the MTA 100 and 400 μmol/L groups and decreased in the MTA 200 μmol/L group; (B) the green fluorescence intensity of each intervention group was compared with that of the model group, and it was increased after the intervention of 400 μmol/L MTA; (C) the percentage of red and green fluorescence in each group; (D) compared with the model group, the relative ratios of red and green fluorescence increased in MTA 100 μmol/L and MTA 400 μmol/L groups, while in MTA 200 μmol/L group showed a decrease. Analysis of variance revealed significance; (E–L) red and green fluorescence photos of different groups. Where (E, F) the dyeing result of the NC group; (G, H) the dyeing result of the MTA 100 μmol/L group, (I, J) the dyeing result of the MTA 200 μmol/L group, and (K–L) the dyeing result of the MTA 400 μmol/L group. The results indicateed that the mitochondrial membrane potential of fat model cells had decreased; the 400 μmol/L MTA intervention increased the mitochondrial membrane potential of the cells. (M) The detection results of ATP concentration after the intervention of obese adipocytes with different concentrations of MTA, and 200 μmol/L MTA slightly reduced it (*p* < 0.05 Vs. model), others unchanged. (N) ROS staining: All MTA groups shifted right (minimal differences, B530‐H: 8.00E5–8.09E5), suggesting no significant ROS increase.

JC‐1 is an ideal fluorescent probe widely used to detect mitochondrial membrane potential, which can assess the membrane potential of cells, tissues, or purified mitochondria. When the mitochondrial membrane potential is high, JC‐1 aggregates in the matrix of mitochondria to form JC‐1 aggregates, which can produce red fluorescence. When the mitochondrial membrane potential is low, JC‐1 exists as a monomer, which can produce green fluorescence. The ratio of mitochondrial depolarization is usually measured by the relative ratio of red and green fluorescence. The results were shown in Figure 4A–L.

The results indicated that in the model group of obesity model cells, a green fluorescent monomer had appeared, and the green fluorescent monomer was larger than the red fluorescent polymer, which indicated that the mitochondrial membrane potential of obesity model cells had decreased. It showed that 400 μmol/L MTA increased mitochondrial membrane potential (0.13 elevation in JC‐1 red/green ratio, *p <* 0.0001), suggesting that the intervention of 400 μmol/L MTA led to an increase in mitochondrial membrane potential.

#### 4.2.4 ATP Concentration Detection

See Figure 4M for ATP concentration detection results after MTA intervention at different concentrations in obese adipocytes. It could be observed that the ATP concentration decreased slightly when 200 μmol/L MTA was used to intervene in obese adipocytes, and there was a significant difference compared with the model group. There was no significant change in ATP concentration in the other groups.

#### 4.2.5 DCFH‐DA Mitochondrial ROS Fluorescence Detection

The results of ROS staining with different concentrations of MTA intervention were shown in Figure 4N. From the test results, the graphs of MTA groups with different concentrations all moved to the right, and the range of right shift was not large, which was very different from that of the HFD group, and the peak value of graphs was not significant (mean: B530‐h fluctuated between 8.00E5 and 8.09E5), suggesting that reactive oxygen species in adipocytes did not increase significantly after MTA intervention with different concentrations.

### 4.3 Gene Editing and Molecular Docking Verified and Predicted the Key Role of TST Gene and the Binding Site of MTA and TST Protein

#### 4.3.1 Cell Adipogenesis Induction Suggests That TST Gene Plays a Key Role in Cell Adipogenesis

**FIGURE 5:**
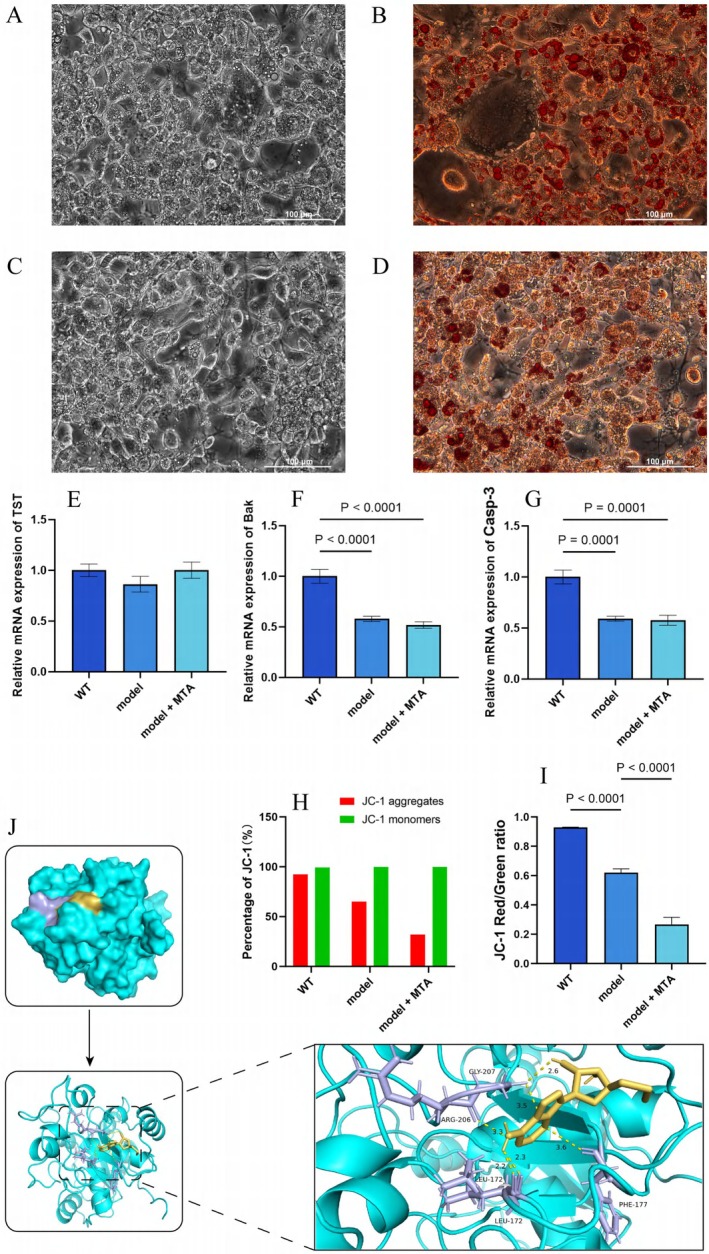
The results of adipogenesis induced by TST knockdown and a visual schematic diagram of molecular docking. Scale: 100 μm. (A, B) Photos of adipogenic‐induced ‐3T3‐L1 wild‐type cells before and after staining under 200× magnification. (C, D) Photos of adipogenic‐induced ‐3T3‐L1‐shRNA#3 cells (TST knockdown) before and after staining. (E–G) RT‐qPCR comparison of key genes after TST silencing: WT (control), model (TST knockdown), and model + MTA (400 μmol/L for 24 h). (H, I) Mitochondrial membrane potential changes via JC‐1 staining: (H) Red/green fluorescence intensity percentages, (I) red/green fluorescence intensity ratios. (J) molecular docking schematic: TST protein (blue) with MTA ligand (yellow) in the active pocket, showing hydrogen bond interactions (purple, e.g., MTA‐LEU‐172, MTA‐GLY‐207), indicating stable binding and supporting the docking simulation.

The staining results showed that there were orange‐red lipid droplets in both cells, which indicated that lipid accumulation existed in both cells, but the size and number of lipid droplets were different. The number of lipid droplets in 3T3‐L1 wild‐type cells was more abundant, and the lipid droplets were larger, which indicated that the lipid accumulation in the cells was more obvious. Compared with 3T3‐L1 wild‐type cells, the lipid droplets in TST knockdown cells were less and smaller, which indicated that the cells still have the ability to accumulate lipid after TST gene knockdown and could continue to induce obesity models, but their lipogenic ability was weakened. The results were shown in Figure 5A–D.

#### 4.3.2 RT‐qPCR Results Suggest That TST Gene Is the Key Link of Apoptosis Induced by MTA

According to the test results of TST gene (Figure 5E), compared with WT group, the expression of TST gene in model group and model + MTA group decreased greatly, and its expression was only about 10% of the control, indicating that the interference efficiency of TST gene in interfering cells reached 90%. According to the results of Bak gene detection (Figure 5F,G), compared with the control group, the expression levels of Bak and Casp‐3 genes in model + MTA cells were 45% and 59% respectively, and the expression levels of Bak and Casp‐3 genes in model + MTA cells were 63% and 86% respectively. The experimental results showed that after TST knockdown, the expression of Bak/Casp‐3 gene in model group decreased, and the expression of Bak gene in model + MTA group decreased slightly, but there was no significant difference with model group. This showed that after TST knockdown, 400 μmol/L MTA intervention failed to cause high expression of Bak and Casp‐3 genes, which was contrary to the previous experimental conclusions.

#### 4.3.3 JC‐1 Staining Suggested That the Recovery of Mitochondrial Function by MTA Disappeared After TST Knockdown

The results showed that compared with WT group, the red fluorescent polymer of model group decreased, the mitochondrial membrane potential decreased and the mitochondrial function was damaged (Figure 5H,I). However, in the model + MTA group, compared with the model group, the red fluorescent polymer continued to decrease, and the relative proportion of red and green fluorescence also decreased, suggesting that the mitochondrial membrane potential of this group of cells continued to decrease compared with the model group. This showed that the mitochondrial function was more seriously damaged after TST knockdown, and the recovery of mitochondrial function by MTA disappeared, contrary to the experimental conclusion before TST knockdown.

#### 4.3.4 Molecular Docking Results Predicted That Hydroxyl Groups of MTA and Amino Acid Residues of TST Formed Hydrogen Bond Interactions

After the docking process, the results and parameters were shown in Table 4.

**TABLE 4:** Molecular docking result parameters.

Mode	Affinity (kcal/mol)	Dist from RMSD lower bound	Best mode RMSD upper bound
1	−6.2	0.000	0.000
2	−6.1	23.031	24.847
3	−6.0	1.832	3.108
4	−5.9	28.765	30.571
5	−5.8	36.217	38.146
6	−5.8	21.954	23.616
7	−5.8	35.845	37.187
8	−5.8	37.345	39.162
9	−5.7	40.403	42.144

*Note:* Affinity (kcal/mol): binding free energy in kcal/mol, indicating the stability of ligand binding to protein. The lower the numerical value, the more stable the combination. Generally, if it is less than −5 or −8, it is considered that the docking is good. It can be seen from the docking results that the affinity of Mode1 is −6.2 kcal/mol, which is the lowest energy mode, which means that the binding of ligand and protein is the most stable in this mode. RMSD: root mean square deviation, which is a measurement method to measure the similarity between two structures. The smaller the RMSD value, the more similar the docking conformation is to the reference conformation, and the higher the accuracy of the docking result may be. Conversely, the reliability of the docking result may be lower. Dist from RMSD lower bound: represents the distance from the lower limit of root mean square deviation. When the RMSD value of the docking result is lower than this lower limit, the conformation is considered to be relatively stable and reliable. Best mode RMSD upper bound, the upper limit of the root means square deviation of the optimal binding mode, that is, the maximum acceptable deviation of the conformation from the reference conformation that the docking program thinks.

Next, we visualized the results in pymol, and the output results were shown in Figure 5. In general, molecular docking simulations were performed to predict the binding mode and affinity between MTA and its target protein TST. The calculated binding free energy for the MTA‐TST complex was −6.2 kcal/mol (the optimal model), which is significantly lower than the conventional threshold (−5.0 kcal/mol), indicating a high binding affinity and strong interaction between MTA and the active pocket of TST.

Further analysis revealed that the hydroxyl group of MTA forms critical hydrogen bonds with several key amino acid residues in TST, including LEU‐172, GLY‐207, and ARG‐206. These residues were likely essential components of the protein's active site, as their interactions with MTA might stabilize the binding complex and contributed to the observed biological effects.

These findings provided structural insights into the molecular basis of MTA's interaction with TST, supporting its potential role in modulating TST‐dependent pathways.

## 5 Discussion

In recent years, there have been more and more studies on fat metabolism. Fat in different parts of the body and different kinds of fat have different functions and exhibit different metabolic mechanisms. Visceral adipose tissue is distributed in the important organs of the abdominal cavity and omentum, and is a component of white adipose tissue. Understanding the relationship between metabolites and visceral white adipose tissue can help us find proper intervention strategies.

Our previous study had verified that the HPRFD diet, as one of the obesity interventions, can change intestinal flora and metabolites. HPRFD can reduce VAT. This study further confirmed that MTA is one of the key differential metabolites affected by diet, which is the focus of our research. It was enriched in visceral adipose tissue of obese mice after HPRFD intervention, which may represent one of the important “material bases” for the diet to reduce visceral adipose volume. Cell experiments have proved that MTA can improve the mitochondrial function of obese adipocytes and promote the apoptosis of these cells.

As one of the important ways of drug discovery, microorganisms and endogenous active substances of humans or animals have always been a subject of concern. The great progress of technology promotes a new strategy of discovering drugs from natural products (Zhang et al. 2020).

As for the metabolite MTA, an endogenous metabolite of the human body, previous research results indicate that its main functions can be summarized as anti‐inflammatory, promoting tumor cell apoptosis, and regulating energy metabolism: (1) Anti‐inflammatory. MTA can affect the function of macrophages in the inflammatory reaction by regulating their polarization state. Specifically, MTA can promote the formation of M2‐type macrophages, thus enhancing its anti‐inflammatory effect. This process involves the interaction between MTA and macrophage surface receptors, which then activate downstream signal pathways, such as NF‐κB and STAT3 pathways. (2) Promoting tumor cell apoptosis and affecting immunity. MTA has been proven to be an effective inhibitor of carboxymethyl transferase in protein as well as a natural by‐product of polyamine biosynthesis. It was reported that MTA can effectively inhibit the growth of certain cells by inducing apoptosis, and the cells treated with MTA show stagnation in the M phase of the cell cycle. The lack of MTA phosphorylase in tumors leads to the accumulation of MTA in the tumor microenvironment, which has a negative impact on the immune function of various immune cells, including T cells and NK cells: MTA interferes with NK cells activating the PI3K/AKT/S6, MAPK/ERK, and NF‐κB pathways downstream of the CD16 receptor (Jacobs et al. 2020). In addition, MTA can further enhance immunosuppression by regulating the secretion of cytokines, such as IL‐10 and TGF‐β (Kerdkumthong et al. 2024). It has also been shown that MTA induces the transcription of matrix metalloproteinases and interleukin‐8 in hepatocellular carcinoma cells in vitro, accompanied by the proliferation and activation of the transcription factor NF‐κB (Kirovski et al. 2011). (3) Affecting energy metabolism. As a precursor in the biosynthesis of several key molecules, MTA plays a significant role in energy metabolism. Previous studies (Lyu et al. 2024) have shown that MTA's role in preventing obesity and insulin resistance may be attributed to its various regulatory activities in energy metabolism, including fatty acid biosynthesis and glucose utilization, which might be regulated by hormones and protein signals with metabolic regulation. For example, MTA inhibits insulin‐induced gene 2 and ELOVL fatty acid elongase 3, while upregulating the mouse β‐2 adrenergic receptor gene and leptin receptor gene (Lyu et al. 2024).

Our study is different from previous findings. We found that the effect of MTA on inducing adipocyte apoptosis is closely related to mitochondrial TST gene. TST is a mitochondrial enzyme. TST gene is one of the few genes expressed in the periphery, which is highly conserved in many organisms and is mainly responsible for coding TST involved in sulfur metabolism. The structure of the gene usually contains multiple functional domains, including a rhodesanase domain related to sulfur transfer. Studies have shown that the expression of TST gene is regulated by many internal and external factors, including the redox state of cells and nutritional conditions. For example, in some bacteria, the expression of TST gene is closely related to the tolerance of cells to cyanide, indicating its importance in coping with environmental stress (De Paula et al. 2020; Zheng et al. 2020). In the past, researchers used the multigene “lean” mouse model to identify that TST gene selectively enhanced mitochondrial function and degraded reactive oxygen species and sulfides. In humans, the expression of TST mRNA in adipose tissue is negatively correlated with the amount of fat in adipose tissue. Therefore, TST gene was identified as a beneficial regulator of mitochondrial function in adipocytes (Morton et al. 2016). This was consistent with the conclusion of this study.

It is worth noting that our research provided the first experimental evidence that MTA may promote cell apoptosis through the mitochondrial pathway. The previous research mentioned above and our research have confirmed that MTA has the potential to become a functional substance.

In general, this study reveals for the first time the molecular mechanism that this high‐protein diet rich in fat activates the apoptosis of VAT mitochondrial cells through the multi‐level regulatory network of “flora‐metabolite‐gene”. Its scientific value is reflected in the following three aspects: (1) Theoretical innovation: the “MTA‐TST‐Bak/Casp‐3” axis is put forward as the key pathway connecting dietary intervention and visceral white adipose tissue remodeling, which breaks through the limitations of the traditional “negative energy balance” theory. (2) Technical integration: The cross‐scale regulation effect of HPRFD was systematically analyzed through the joint analysis of multi‐omics, which provided methodological reference for the study of complex dietary intervention. (3) Transformation potential: Microorganisms, endogenous active substances of human beings or animals, have become an important way of drug discovery (Zhang et al. 2020). As an endogenous adipocyte apoptosis inducer, perhaps MTA can be delivered to white adipose tissue by nano‐delivery system (Liu et al. 2022; Meng et al. 2022) or improved way, so that it can play a targeted biological role in specific tissues and provide a new strategy for the prevention and control of obesity and related metabolic diseases.

Limitations and Future Directions: While this study demonstrates significant progress, several limitations warrant further investigation: (1) Technical Limitations: Due to limited adipose tissue samples and low protein yield, protein‐level validation (e.g., Western blot) was not performed; only RT‐qPCR was used to confirm gene expression changes. (2) Mechanistic Gaps: The precise regulation of TST gene/protein expression by MTA remains unclear and requires advanced techniques for deeper exploration. (3) Microbiota‐MTA Relationship: The potential role of gut microbiota in MTA production is unknown—future studies should clarify whether specific flora directly influence MTA levels. (4) Clinical Translation: Human trials are needed to evaluate MTA's bioavailability, safety, and pharmacological effects.

Future Directions: (1) MTA‐TST Interaction: Investigate protein‐DNA/metabolite/protein interactions to elucidate molecular mechanisms. (2) Microbiota Regulation: Explore strategies to modulate gut flora and fecal microbiota transplantation (FMT) to enhance HPRFD's lipid‐lowering effects. (3) Human Intervention Studies: Conduct population‐based trials with HPRFD, analyzing blood/fecal metabolites (including MTA) to bridge preclinical and clinical research. (4) Cellular Effects Beyond Adipocytes: Examine MTA's impact on hepatocytes, islet cells, and muscle cells, alongside its role in adipocyte apoptosis. (5) Adipose Tissue Specificity: Assess differential MTA responses in subcutaneous vs. VAT.

## 6 Conclusion

Generally speaking, a high‐protein diet regulates fecal and VAT metabolism and metabolite MTA, and then activates the VAT mitochondrial related apoptosis pathway (TST‐Bak/Casp‐3) to reduce VAT and exert an anti‐obesity effect. As a core metabolite, MTA has the potential to develop functional substances.

## Author Contributions

**Dan Yan:** conceptualization (supporting), funding acquisition (lead), resources (lead), supervision (supporting). **Xinli Yang:** conceptualization (lead), data curation (lead), formal analysis (lead), investigation (lead), methodology (lead), project administration (lead), resources (lead), software (lead), supervision (lead), validation (lead), visualization (lead), writing – original draft (lead), writing – review and editing (lead). **Jianglan Long:** conceptualization (supporting), formal analysis (supporting), funding acquisition (supporting), supervision (supporting), writing – review and editing (supporting). **Aiting Wang:** formal analysis (supporting), supervision (supporting), writing – review and editing (supporting). **Zhe Shi:** formal analysis (supporting), writing – review and editing (supporting). **Yueyue Wang:** formal analysis (supporting), writing – review and editing (supporting).

## Conflicts of Interest

The authors declare no conflicts of interest.

## Supporting information


**Table S1.** The common differential metabolic pathways and differential genes and metabolites in each pathway between groups.

Supplementary material S2.

## Data Availability

The data that support the findings of this study are available on request from the corresponding author.
