# Supplementary material for: MTA‐TST Axis‐Mediated Apoptosis Activation: A Multi‐Omics Insight Into High‐Protein Diet's Anti‐Adiposity Effect
Source: Food Sci Nutr. 2025 Jul 9;13(7):e70511. doi: 10.1002/fsn3.70511 (PMC12238778; doi:10.1002/fsn3.70511)
Supplement: Supplementary file 1 — Table S1. The common differential metabolic pathways and differential genes and metabolites in each pathway between groups. [file FSN3-13-e70511-s002.docx]

**Supplementary TABLE 1 | The common differential metabolic pathways and differential genes and metabolites in each pathway between groups**

| **common differential metabolic pathways between groups** | **NC vs HFD** | **HFD vs HPRFD** |
| --- | --- | --- |
|  | **Gene/Metabolite** | **Gene/Metabolite** |
| Galactose metabolism | ENSMUSG00000025877 | ENSMUSG00000025877 |
|  | ENSMUSG00000032401 | ENSMUSG00000032401 |
|  | Dulcitol galactitol，dulcite | Dulcitol galactitol，dulcite |
|  |  |  |
| Cysteine and methionine metabolism | ENSMUSG00000030268 | ENSMUSG00000030268 |
|  | ENSMUSG00000044986 | ENSMUSG00000044986 |
|  | 5'-Deoxy-5'-(Methylthio)Adenosine | 5'-Deoxy-5'-(Methylthio)Adenosine |
|  |  |  |
| Arachidonic acid metabolism | ENSMUSG00000003484 | ENSMUSG00000003484 |
|  | ENSMUSG00000029919 | ENSMUSG00000029919 |
|  | ENSMUSG00000029925 | ENSMUSG00000029925 |
|  | ENSMUSG00000030483 | ENSMUSG00000030483 |
|  | ENSMUSG00000051855 | ENSMUSG00000051855 |
|  | Prostaglandin F2alpha | Prostaglandin D2 |
|  |  |  |
| Biosynthesis of unsaturated fatty acids | ENSMUSG00000021228 | ENSMUSG00000021228 |
|  | ENSMUSG00000042540 | ENSMUSG00000042540 |
|  | Eicosapentaenoic acid | Eicosapentaenoic acid |
