# Supplementary material for: MTA‐TST Axis‐Mediated Apoptosis Activation: A Multi‐Omics Insight Into High‐Protein Diet's Anti‐Adiposity Effect
Source: Food Sci Nutr. 2025 Jul 9;13(7):e70511. doi: 10.1002/fsn3.70511 (PMC12238778; doi:10.1002/fsn3.70511)
Supplement: Supplementary file 2 — Supplementary material S2. [file FSN3-13-e70511-s001.docx]

**Supplementary material 2 | Adipogenic Induction of T3-L1 Cell Line**

A microscope was used to observe the results of adipogenic staining, and the images were collected and evaluated. When the induction is successful, the lipid droplets will appear red or orange after combining with oil red O dye. The induction process and the results of oil red O staining after induction are as follows:


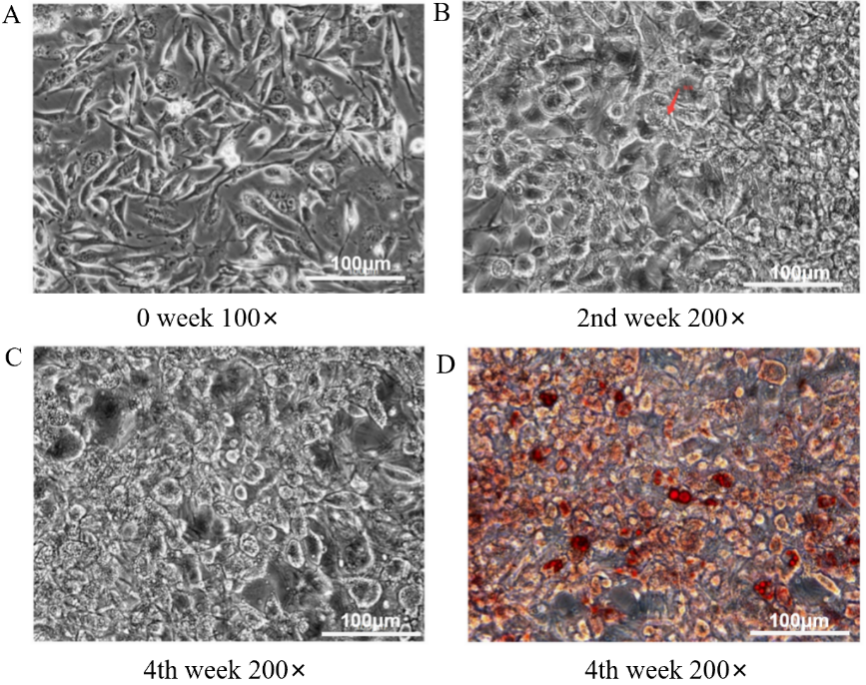


**Supplementary FIGURE 1 | The cells were observed under 200 times eyepiece in the second week and the fourth week of induction.** (A) cell condition under the field of vision of 100× eyepiece before adipogenesis induction; (B-C) cell status after 2 weeks and 4 weeks of adipogenesis induction; (D) showed the cell state after oil red O staining after 4 weeks of induction. The scale is 100 μm.Based on the proportion of lipid droplets and the size of lipid droplets, we observed that the number of lipid droplets increased significantly in the late stage of induction, and the size of lipid droplets no longer increased significantly, which judged that lipid induction reached an ideal state.
